# Combination treatment with triptolide and hydroxycamptothecin synergistically enhances apoptosis in A549 lung adenocarcinoma cells through PP2A-regulated ERK, p38 MAPKs and Akt signaling pathways

**DOI:** 10.3892/ijo.2015.2814

**Published:** 2015-01-07

**Authors:** GUANMIN MENG, WEI WANG, KEQUN CHAI, SUWEN YANG, FANGQIONG LI, KAI JIANG

**Affiliations:** 1 Department of Clinical Laboratory, Tongde Hospital of Zhejiang Province, Hangzhou 310012, P.R. China; 2 Department of Clinical Laboratory, Sir Run Run Shaw Hospital, Medical College, Zhejiang University, Hangzhou 310016, P.R. China

**Keywords:** triptolide, hydroxycamptothecin, apoptosis, protein phosphatase 2A, mitogen-activated protein kinases, Akt

## Abstract

Lung cancer is the leading cause of cancer death worldwide. Recently, two plant-derived drugs triptolide (TP) and hydroxycamptothecin (HCPT) both have shown broad-spectrum anticancer activities. Our previous study documented that combination treatment with these two drugs acted more effectively than mono-therapy, however, the molecular basis underlying the synergistic cytotoxicity remains poorly understood. In this study, we aimed to clarify the molecular mechanism of TP/HCPT anticancer effect in A549 lung adenocarcinoma cells, by investigating the involvement of phosphatase 2A (PP2A) and PP2A-regulated mitogen-activated protein kinases (MAPKs) and Akt signaling pathways. The results showed that TP and HCPT synergistically exerted cytotoxicity in the growth of A549 cells. Combinatorial TP/HCPT treatment significantly enhanced the activation of caspase-3 and -9, Bax/Bcl-2 ratio, release of cytochrome *c* from mitochondrial and subsequent apoptosis. While the Akt survival pathway was inhibited, ERK and p38 MAPKs were dramatically activated. Furthermore, the activity of PP2A was significantly augmented. Regulation of p38, ERK and Akt by PP2A was demonstrated, by using a specific PP2A inhibitor okadaic acid (OA). Finally, pharmacological inhibitors OA, SB203580, SP600125 and PD98059 confirm the role of PP2A and its substrates ERK, p38 MAPK and Akt in mediating TP/HCPT-induced apoptosis. Taken together, this study provides the first evidence for a synergistic TP/HCPT anti-cancer activity in A549 cells and also supports a critical role of PP2A and PP2A-regulated signaling pathways, providing new insight into the mode of action of TP/HCPT in cancer therapy.

## Introduction

Lung cancer is one of the most common cancers in the world and accounts for ~28% of all cancer deaths, and non-small cell lung cancer (NSCLC) accounts for ~80% of lung cancers (1). Currently, surgery, chemotherapy and radiotherapy are still the main conventional treatment of lung cancer. While chemotherapy significantly improves symptoms and the quality of life of patients with lung cancer, however, its efficacy and safety remain a primary concern as toxicity and other side effects of chemotherapy remains a key obstacle (2). Therefore, there is an urgent need for novel strategies or reagents of lung cancer. Alternative medicines, especially herbal therapies (3,4), are becoming more and more attractive. Among these therapies, traditional Chinese medicine (TCM) is probably the best established. Recently, to discover and develop specific herbal extracts and combinations formulas in TCM that can preferentially kill cancer cells without significant toxicity has become an important area in lung cancer therapy.

**Figure 1:**
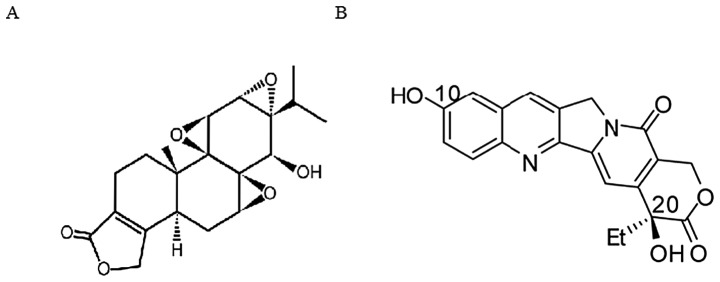
Chemical structures of the compounds. (A) Triptolide (TP). (B) 10-hydroxycamptothecin (HCPT).

Triptolide (TP), a diterpenoid triepoxide compound (Fig. 1A), is one of the major biologically active components extracted originally from the Chinese herb *Tripterygium wilfordii* Hook F (5). Numerous studies have revealed that TP has a myriad of biological properties, including immunosuppression, anti-inflammation, and has been applied to the treatment of autoimmune diseases such as nephritis and rheumatoid arthritis (6). TP has been reported to exert anti-cancer activity in diverse tumor cell types *in vitro*, including colon cancer (7), ovarian cancer (8), myeloma (9), myeloid leukemia (10), adrenal cancer (11) and pancreatic cancer cells (12), and to prevent tumor growth *in vivo* via inhibiting cell proliferation and inducing apoptosis (13). Therefore, with its broad-spectrum anticancer activity, TP has a considerable potential as a chemotherapeutical agent.

The natural product camptothecin (CPT) is a pentacyclic alkaloid, first isolated in 1966 from the extract of a Chinese plant *Camptotheca accuminata*. CPT and its analogues have received increasing attention as a promising class of antitumor agents, which act by a unique mechanism inhibiting DNA topoisomerase (14). Among the CPT family, 10-hydroxycamptothecin (HCPT), as shown in Fig. 1B, displays more potent antitumor effects and is less toxic in experimental animals and in human clinical evaluations as compared with CPT. Due to its special cell killing mechanism, that is different from that of other cytotoxic agents, it is difficult to induce cross drug resistance when HCPT is applied together with other anticancer agents. Similarly to TP, HCPT also shows a broad spectrum of antitumor activity against various types of cancers, such as gastric carcinoma, hepatoma, leukemia, bladder carcinoma, and lung cancer (15–18).

A wealth of data now implicate that TP in combination with other anticancer agents, such as idarubicin (19), sorafenib (20), oxaliplatin (7), 5-FU (21), AraC (22), TRAIL (23) and ionizing radiation (24), synergistically increase their cytotoxic effects, suggesting that, next to its potential as a single drug, TP also appears to have potential as a combinatorial drug for the treatment of malignancies. Similarly to the aforementioned findings, our previous study demonstrated that TP combined with HCPT can synergistically suppress the proliferation of pancreatic cancer cell line PANC-1, and induces cell apoptosis (25). However, the detailed mechanism for the synergistic effect of these two herbal medicines remains unclear. The present study was designed to determine the combined efficacy of TP and HCPT on A549 human lung adenocarcinoma cells, and to investigate whether the synergistic cytotoxicity on cell growth may be attributed to induction of apoptosis. Furthermore, to uncover the molecular mechanisms, the involvement of mitochondria-dependent biochemical markers, as well as tumor suppressor protein phosphatase 2A (PP2A), Akt and MAPKs signaling pathways in mediating TP/HCPT-triggered apoptosis were investigated to help us to better use TCMs in cancer therapy.

## Materials and methods

### Chemicals

Triptolide (≥98%) was obtained from Beijing Fan-China Biotechnology Co. Ltd. (Beijing, China). 7-ethyl-10-hydroxycamptothecin (HCPT) was obtained from Sigma (NY, USA). These drugs were stored in a stock solution in dimethylsulfoxide (DMSO) at −20°C and diluted to various concentrations with serum-free culture medium when used. Okadaic acid (OA) was purchased from Sigma-Aldrich (St. Louis, MO, USA). SB203580, PD98059 and LY294002 were obtained from Calbiochem (San Diego, CA, USA). The 3-(4.5-dimethylthiazol-2-yl)-5-(3-carboxymethoxyphenyl)-2- (4-sulfophenyl)-2H-tetrazolium, inner salt (MTS) was from Promega Co. Ltd. (Southampton, UK), and phenazine methosulfate (PMS) (1×10^−4^ mol/l) was purchased from Sigma. Annexin V/FITC apoptosis detection kit was obtained from BD Pharmingen (San Diego, CA, USA). Serine/threonine phosphatase assay kit was purchased from Promega (Madison, WI, USA). Mitochondria/cytosol fractionation kit was from Biovision (Mountain View, CA, USA). Antibodies against caspase-3, -9, cytochrome c, Bax, Bcl-2, PP2A-A subunit, PP2A-C subunit, Akt and all MAPK family (ERK1/2, p38 and JNK) were all obtained from Cell Signaling Technology (Beverly, MA, USA). Antibodies against caspase-12, GRP78 and CHOP were purchased from Santa Cruz Biotechnology (Santa Cruz, CA, USA). Anti-phospho-PP2A Cα (Tyr307) was from Abcam (Cambridge, MD, USA). Antibodies against β-actin and all of the secondary antibodies were obtained from Kangcheng (Shanghai, China).

### Cell culture

Human lung cancer A549 cells (American Type Culture Collection; ATCC CCL185) were maintained in monolayer culture at 37°C in a humidified atmosphere with 5% CO_2_ in RPMI-1640 medium (Gibco-BRL, USA) supplemented with 10% fetal bovine serum (FBS) (Sijiqin Biotechnology Co. Ltd., China), 1% penicillin-streptomycin (100 U/ml penicillin and 100 μg/ml streptomycin).

### Cytotoxicity assay

To determine the optimal concentration of the combination of TP and HCPT, A549 lung cancer cells (1×10^4^ cells/well in 96-well plates) were exposed to increasing concentrations of TP (0, 6.25, 12.5, 25, 50, 100, 200 and 400 ng/ml) or HCPT (0, 0.25, 0.5, 1, 2, 4, 8 and 16 μg/ml) for 24 h, respectively. For the cytotoxicity assay of TP/HCPT combination treatment, A549 cells were treated with TP (25 ng/ml) and variable concentrations of HCPT (0, 0.5, 1, 2, 4 and 8 μg/ml) for 24 h, and cell viability was evaluated by the MTS assay using tetrazolium compound MTS and the electron coupling reagent PMS. After incubation for the indicated time, 20 μl MTS/PMS mixture was added to each well; then, the cells were incubated for 1 h and absorbance was measured at 490 nm. The background absorbance from the control wells was subtracted from the actual absorbance value. Three duplicate studies were performed for each experimental condition. Dose-response curves were plotted on the basis of the data derived from the MTS assay.

### Combination index and dose reduction index analyses

The multiple drug effect analysis of Chou and Talalay (26), which is based on a median-effect principle, was used to evaluate the nature of the interaction between TP and HCPT. The combination index (CI) is a parameter that indicates whether the interaction of two or more drugs is synergistic, additive, or antagonistic. Briefly, synergism, additivity and antagonism are indicated by CI<1, CI=1 and CI>1, respectively. The dose reduction index (DRI) is a parameter that indicates the degree to which a drug dose can be reduced when used in combination with another drug and maintain an equivalent effect level. The CI and DRI for TP/HCPT were calculated based on the data from the MTS assay as described previously (25,27).

### Determinations of cell morphology and cell apoptosis

A549 cells (1.5×10^5^ cells/well) were seeded in 6-well culture plates for 24 h and then were treated with TP and HCPT alone or in combination for 24 h, and then photographed under phase-contrast microscope and harvested by centrifugation. For apoptosis determination, we used the Annexin V/FITC kit as described by the manufacturer. The collected cells were resuspended in binding buffer at a concentration of 1×10^6^ cells/ml. After incubation, 100 μl of the solution was added by 5 μl of Annexin V-FITC and 5 μl of propidium iodide (PI), and then incubated for 15 min at room temperature in the dark. At the end of incubation, 400 μl of binding buffer was added, and the cells were analyzed immediately by flow cytometry. Flow cytometry analysis was performed with untreated cells as a control.

### Western blot analysis

Total protein extracts were prepared as described previously (28). Cells were suspended in cell lysis buffer containing 150 mM NaCl, 50 mM Tris (pH 7.6), 15 mM EDTA, 1 mM phenylmethylsulfonyl fluoride, 1 mM Na_3_VO_4_, and 1 mM NaF, and complete protease inhibitor tablet (Roche, Indianapolis, IN, USA) on ice for 30 min. The lysate solution was then centrifuged at 20,000 g for 20 min at 4°C, and the supernatant protein extract was stored at −80°C.

For cytosolic cytochrome *c* analysis, cytosolic fractions were prepared by using Mitochondria/Cytosol fractionation kit according to the manufacturer’s instructions. Briefly, the cells were collected and suspended in cytosolic extraction buffer containing 0.1% DTT and protease inhibitor on ice for 10 min. The mixture was homogenized in an ice-cold dounce tissue grinder (40–50 strokes) and centrifuged at 700 g for 10 min. The supernatants were then transferred into fresh tubes, centrifuged at 10,000 g for 30 min, and the supernatants were collected as cytosolic fraction and stored at −80°C.

The concentration of protein in each cell lysate was determined by using a BCA protein assay kit (Pierce, Rockford, IL, USA) with bovine serum albumin (BSA) as the standard. Subsequent western blot analysis was performed as described (reference?) previously. All blots were developed using enhanced chemoluminescence reagents (Super signal dura kit, Pierce) following the manufacturer’s instructions.

### PP2A phosphatase activity assay

PP2A activity was determined using a serine/threonine phosphatase assay system in accordance with the manufacturer’s protocols. Cells were briefly lysed with a phosphatase lysis buffer (50 mM Tris-HCl pH 7.5, 10% glycerol, 0.05% β-mercaptoethanol, 0.1 mM EDTA, 0.05% Triton X-100, 0.5 mM PMSF, phosphatase inhibitor cocktail) and measured for phosphatase activity using a PP2A-type specific buffer (250 mM imidazole pH 7.2, 1 mM EGTA, 0.1% β-mercaptoethanol, 0.5 mg/ml bovine serum albumin). Free phosphate, generated from a synthetic phosphothreonine peptide RRA(pT)VA specific for PP2A, was quantified by measuring molybdate/malachite green/phosphate complex at 630 nm. EGTA and EDTA were included in the lysis buffer to inhibit PP2B and PP2C, respectively. The effective range of the assay is 100–4,000 pmol of phosphate.

### Statistics

Results are expressed as mean ± SE. Statistical significance between groups was determined using one-way ANOVA and Dunnett’s comparison. p<0.05 were considered statistically significant.

## Results

### Combination treatment of TP and HCPT induced growth inhibition of A549 cells

**Figure 2:**
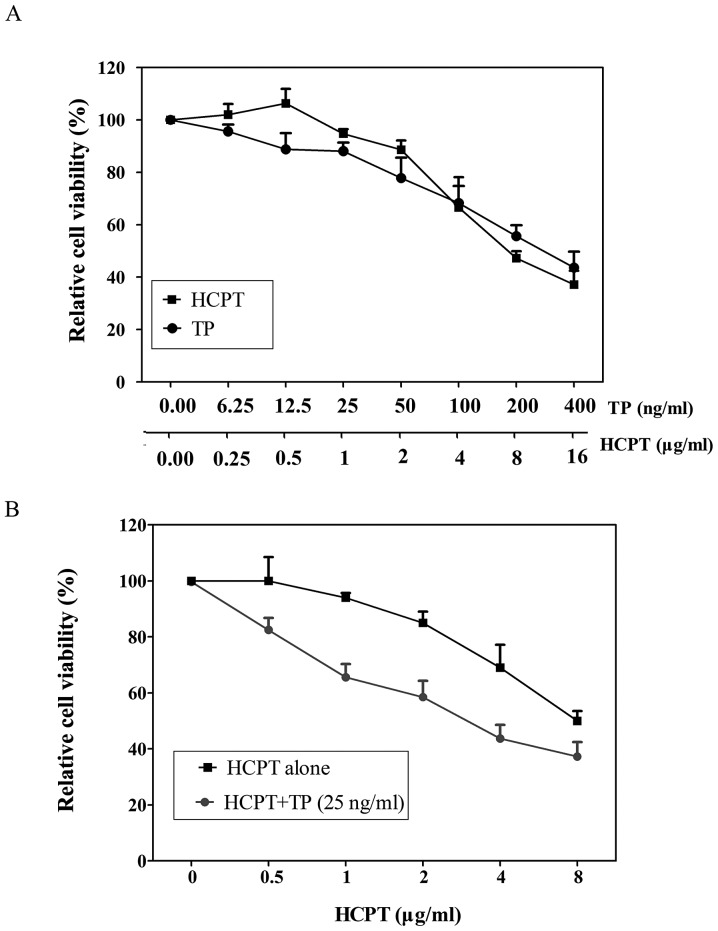
Combinatorial effects of TP and HCPT on A549 cells. (A) Individual effect of TP or HCPT on cellular growth. A549 cells were treated with graded concentrations of TP (6.25, 12.5, 25, 50, 100, 200 and 400 ng/ml, respectively) or HCPT (0.25, 0.5, 1, 2, 4, 8 and 16 μg/ml, respectively) for 24 h. Cellular growth was measured by MTS assay. Data are expressed as the means ± standard error (SE) (n=4). (B) The combined effects of TP and HCPT on cellular growth. A549 cells were treated with HCPT (0–8 μg/ml) combined with or without TP (25 ng/ml) for 24 h, and the cell proliferation was monitored. Data are expressed as the means ± SE (n=4).

The effect of TP or HCPT as a single agent on the growth of the A549 lung adenocarcinoma cells was first assessed. As shown in Fig. 2A, either TP or HCPT individually caused a markedly dose-dependent reduction in cell viability, with 50% growth inhibition (IC_50_) of 273.2 ng/ml and 8.62 μg/ml, respectively. Next, we adopted a combination treatment by keeping the concentration of TP constant at IC_10_ value (25 ng/ml), together with varying concentrations of HCPT (0–8 μg/ml). We found that the combined treatment of TP and HCPT (TP/HCPT) substantially suppressed A549 cell growth as compared to single drug alone. Fig. 2B showed a 3.7-fold decrease of IC_50_ for HCPT when added in presence of 25 ng/ml TP (IC_50_=2.34 μg/ml), compared to HCPT treatment alone, indicating that the combination treatment with TP and HCPT is more effective in inhibition of A549 cell growth than single drug treatment.

In order to assess their synergistic effect on A549 cells, we evaluated synergy using CalcuSyn software to evaluate the combination index (CI) which was originally described by Chou and Talalay (26,27), when synergism, additivity and antagonism are defined by CI<1, CI=1 and CI>1, respectively. The CI value was nearly 1.20 when A549 cells were exposed to the combination of 25 ng/ml TP and 0.5 μg/ml HCPT, indicating a slight antagonism. However, when A549 cells were exposed to the combination of 25 ng/ml TP and variable concentrations of HCPT (1–8 μg/ml), it demonstrated clear evidence of synergy since the CI value ranged from 0.4 to 0.7 (<1) (Table I). Similar to our recent findings in pancreatic cancer cell line PANC-1 (25), a higher synergistic effect (lower CI value) was observed for lower dose combination. Once the interaction between the two agents was found to be synergistic, we next sought to determine the dose reduction index (DRI) values for TP and HCPT in A549 cancer cells. The DRI analysis further indicated that TP/HCPT combination has the potential to reduce both the doses of TP (ranging from 1.10- to 20.97-fold dose reduction) and HCPT (ranging from 1.60- to 3.74-fold dose reduction) in A549 cells (Table I). In all the subsequent experiments, we treated the cells with the combination of TP (25 ng/ml) and HCPT (1–4 μg/ml) and TP/HCPT exhibited higher synergistic effect in inhibiting the growth of A549 lung adenocarcinoma cells.

### Treatment with TP and HCPT synergistically induced apoptosis of A549 cells

**Figure 3:**
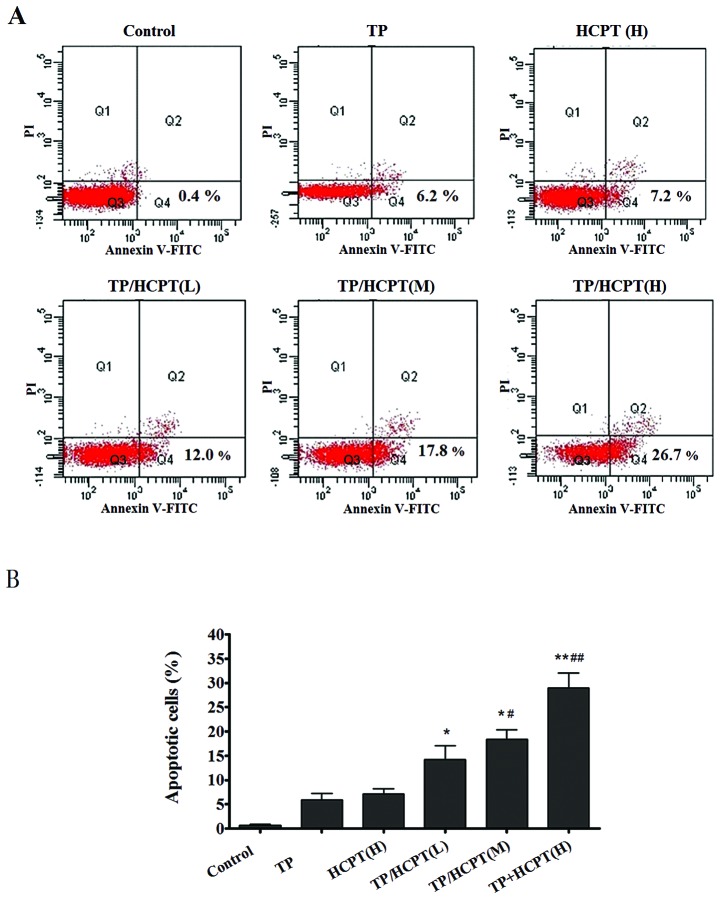
Effect of TP and HCPT combination treatment on the induction of apoptosis. (A) Flow cytometry analyses of A549 cells under various treatments using double staining with Annexin V (horizontal line) and propidium iodide (PI, vertical line); TP, 25 ng/ml TP; HCPT(H), 4 μg/ml HCPT; TP/HCPT(L), 25 ng/ml TP + 1 μg/ml HCPT; TP/HCPT(M), 25 ng/ml TP + 2 μg/ml HCPT; TP/HCPT(H), 25 ng/ml TP + 4 μg/ml HCPT. (B) Bar graphs representing the proportion of early stage apoptosis cells (Annexin V-positive/PI-negative). Data are expressed as the means ± SE (n=3). ^*^p<0.05 and ^**^p<0.01 vs. TP group; ^#^p<0.05 and ^##^p<0.01 vs. HCPT group.

To investigate the effect of TP/HCPT on apoptosis in A549 cells, Annexin V/propidium iodide (PI) staining-based FACS analysis was performed to detect the externalization of phosphatidylserine on the cell membrane, a hallmark of early apoptosis. Cells undergoing early-stage apoptosis are stained with Annexin V-FITC-positive and PI-negative. Fig. 3A quantifies the increase in apoptotic cells labeling with Annexin V^+^/PI^−^, which increases from 0.4% in the control group to 6.2 and 7.2% in the TP- and HCPT-treated group, respectively. Various combinatorial treatments resulted in more apoptotic cells than either single-drug treatment alone, by increasing the apoptosis rate up to 12, 17.8 and 26.7%. It revealed that various combination treatments induced significantly higher percentage of apoptosis as compared to either TP or HCPT treatment alone (Fig. 3B), indicating TP/HCPT can promote apoptosis.

### Synergistic effect of TP and HCPT on cell apoptotic pathways

**Figure 4:**
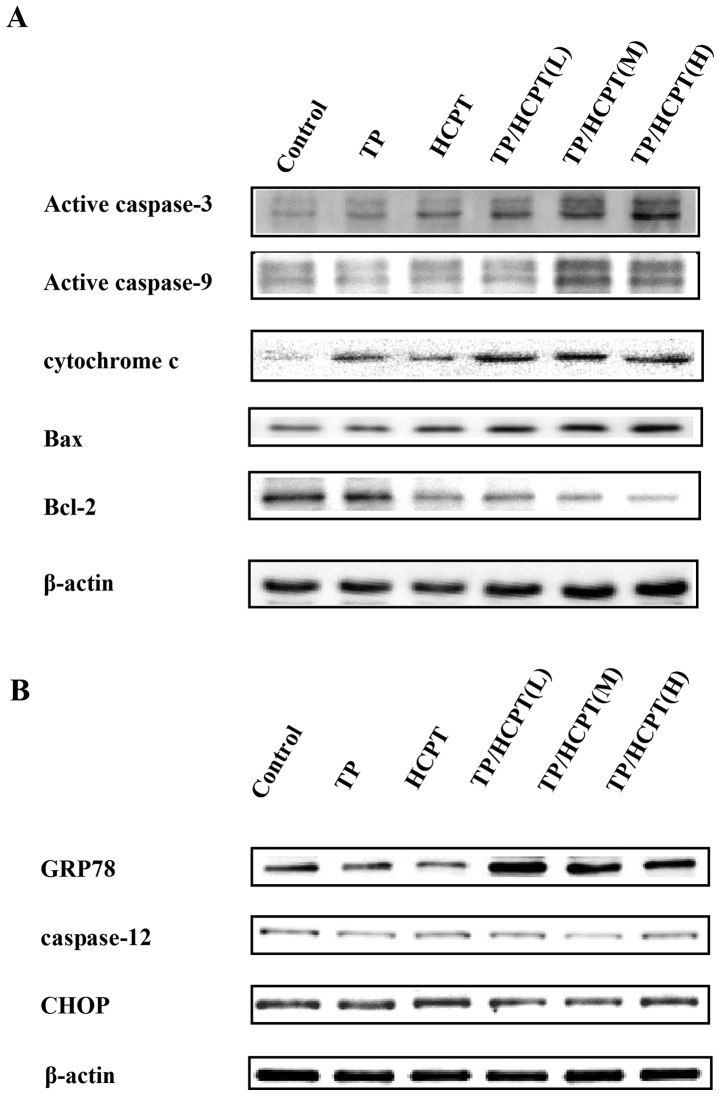
Effect of TP/HCPT treatment on the apoptosis-associated proteins in A549 cells. A549 cells under various treatments were harvested for examining the associated protein levels. (A) Mitochondrial apoptotic pathway detected by antibodies against caspase-3, -9, cytochrome *c*, Bax and Bcl-2. (B) ER stress apoptotic pathway detected by antibodies against caspase-12, GRP78 and CHOP. β-actin was used as an internal control.

In order to determine whether TP/HCPT induced apoptosis is mediated via the mitochondria-dependent and/or the ER stress-triggered signaling pathways, we examined the effects of TP/HCPT on the related protein level changes by western blot assay. Initially, the expression of caspase-9, -3, cytochrome *c* (cytosolic), Bax and Bcl-2 were examined to evaluate the mitochondrial apoptotic pathways. Caspase-9 is known to be involved in the activation of the caspase cascades for cleaving and activating caspase-3, which is an integral step in most apoptotic events. The results indicated that TP or HCPT each only resulted in a slight upegulation of active (cleaved) caspase-3 and -9 production, whereas the combinatorial TP/HCPT treatment caused pronounced upegulation of active caspase-3 and -9 (Fig. 4A), suggesting that enhanced cell apoptosis is mediated through activation of caspase-3 and -9 pathway. In addition, the combined treatment also augmented the release of cytochrome *c* from mitochondria, diminution of Bcl-2, and marked increase in Bax expression (Fig. 4A), which displayed features of mitochondria-dependent apoptotic signals.

Next, we further determined the expression of three hallmarks of endoplasmic reticulum stress (ERS), including glucose regulated protein 78 (GRP78) and CHOP which are known to promote ER stress-induced apoptosis (29,30), as well as ER-specific caspase-12 (31). It revealed that TP/HCPT combination treatment upegulated the GRP78 expression, which is a key regulator in ER stress signaling that has a dynamic capacity to regulate the balance between cell survival and apoptosis (Fig. 4B). However, CHOP and caspase-12, the critical executioners involved in the ER stress-mediated apoptosis, were not affected by the treatments. These results suggest that the apoptotic action of TP/HCPT is mainly mediated by caspase-9 and -3 mitochondrial apoptotic pathways.

### Synergistic effect of TP and HCPT on PP2A activity as well as MAPKs and Akt signaling pathways

**Figure 5:**
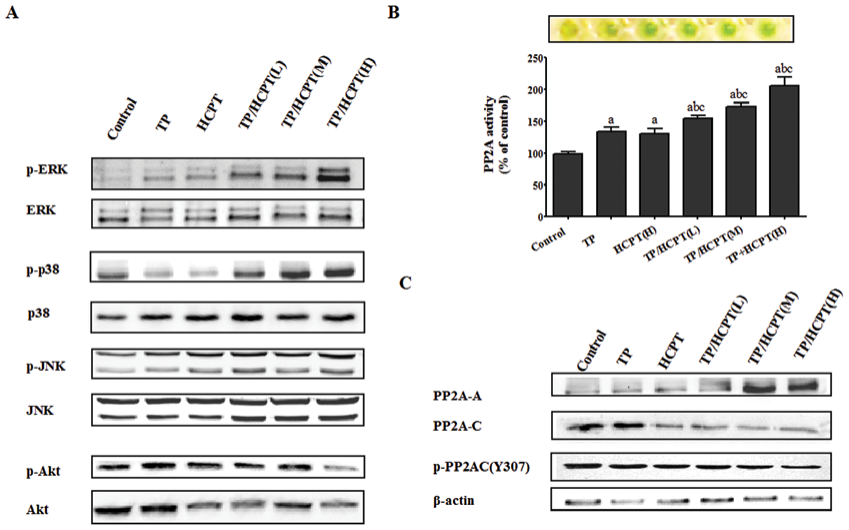
Effect of TP/HCPT treatment on the MAPKs and Akt signaling pathways as well as PP2A activity in A549 cells. (A) The cell lysates from A549 cells under various treatments were subjected to immunoblot analysis with antibodies that detect either the specified protein or the specified protein when it is phosphorylated at a designated site, which are representative of at least 3 separate experiments. (B) Phosphatase activity of PP2A was determined with phosphatase assay system (see Materials and methods). The values are expressed as a percentage of the control (untreated cells; 100% PP2A activity). a, ^*^p<0.05 vs. the control group; b, ^*^p<0.05 vs. TP group; c, ^*^p<0.05 vs. HCPT group (n=4). (C) Alterations of various PP2A subunit levels detected by antibodies against the PP2A-A subunit, PP2A-C subunit and phospho-PP2A Cα (Tyr307). β-actin was used as an internal control.

Multiple signaling pathways, such as mitogen-activated protein kinases (MAPKs) family and protein kinase B (Akt) signal transduction pathways, are essential for cell survival and important for the regulation of apoptosis (32–34). To begin elucidating the signaling events that mediate TP/HCPT-induced apoptosis, we first analyzed the activation of three MAPK effectors (p38, JNK and ERK1/2) and Akt in A549 cells using phospho-specific antibodies. As shown in Fig. 5A, TP/HCPT treatment synergistically evoked a dramatic phosphorylation of ERK1/2 and p38 (i.e., activation), in a similar pattern that a maximum response occurred when TP was combined with HCPT at the highest concentration (4 μg/ml). However, no significant effect of TP/HCPT combination on JNK phosphorylation was observed. The levels of non-phosphorylated p38, JNK and ERK were unaffected by either mono-therapy or combinatorial treatment. On the other hand, we observed that while the phosphorylated Akt was slightly stimulated by TP alone, however, combination treatment of TP and HCPT significantly inhibited TP-induced activation of Akt (Fig. 5A). In particular, cells exposed to TP combined with the highest concentration of HCPT underwent remarkable decrease in Akt phosphorylation.

Protein phosphatase 2A (PP2A) is one of the major protein serine/threonine phosphatases that regulate diverse cellular functions such as cell division and transcription (35), and has attracted considerable attention due to its apoptosis-inducing effect and tumor-suppressing function (36–38). Hence, we evaluated the contribution of PP2A by accessing the phosphatase activity, and the levels and modification of PP2A composition subunits. We noted that the activity of PP2A was stimulated by both TP and HCPT mono-therapies. Moreover, combinatorial TP/HCPT treatment markedly enhanced the activity of PP2A in a dose-dependent manner, with the maximum response occurring when TP was combined with the highest concentration of HCPT (Fig. 5B). Furthermore, alteration of PP2A composition subunit levels was observed. As shown in Fig. 5C, structural A subunit was dramatically enhanced by TP/HCPT combination treatment. In contrast, TP/HCPT caused a marked decrease in the level of total PP2A-C. Subsequently, the phosphorylation at Tyr307 (Y307) of the PP2A-C subunit was investigated because it contributes to decrease in PP2A activity (39). It was noteworthy that TP/HCPT treatment induced the downregulation of PP2Ac (Y307) phosphorylation, which was in accordance with PP2A activation.

### TP/HCPT induced apoptosis is mediated through PP2A-regulated ERK, p38 MAPK and Akt signaling pathways

**Figure 6:**
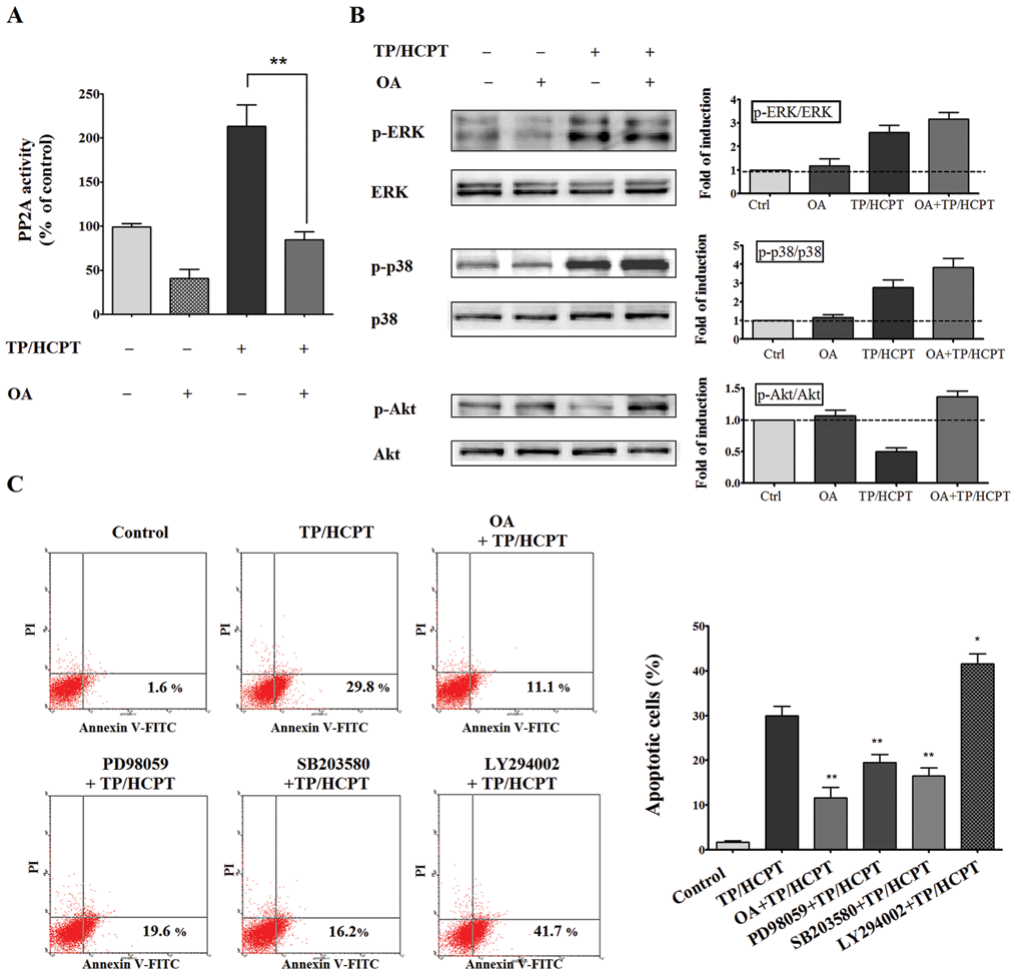
Effect of PP2A inhibition on ERK, p38 and Akt signaling pathways, as well as TP/HCPT-induced apoptosis. (A) TP/HCPT-enhanced PP2A activity was inhibited by OA. A549 cells were also treated with OA (50 nM) for 2 h prior to 24 h of combinatorial treatment with TP (25 ng/ml) and HCPT (4 μg/ml). Cell lysates were prepared and assayed for phosphatase activity of PP2A. The values are expressed as a percentage of the control (untreated cells; 100% PP2A activity). ^**^p<0.01 vs. the TP/HCPT group (n=3). (B) Effect of PP2A inhibition on ERK, p38 and Akt signaling pathways. Cell lysates after different treatments were prepared and subjected to western blot analysis. The results are representative of three independent experiments, and the corresponding densitometric analyses for relative protein expression were shown in the right hand panels. (C) Effect of inhibitors of pp2A, p38, ERK and Akt on TP/HCPT-induced apoptosis. The cells were treated with OA (50 nM), p38 inhibitor-SB 203580 (10 μM), ERK inhibitor-PD 98059 (10 μM) and Akt inhibitor-LY294002 (25 μM) for 2 h prior to 24 h of TP/HCPT treatment. Then the cells were collected and analyzed using Annexin V/PI double staining, and bar graphs shown in the right hand panel representing the apoptotic rates. ^*^p<0.05 and ^**^p<0.01 vs. the TP/HCPT group (n=3).

PP2A has been demonstrated to act as a deactivator of several kinases as its substrates, such as MAPKs and Akt (40–42). To confirm this role, we used okadaic acid (OA), a selective inhibitor of PP2A, and to examine whether inhibition of PP2A by OA modulates the phosphorylation status of ERK1/2, p38 MAPKs and Akt in TP/HCPT-treated A549 cells. Notably, pretreatment of A549 cells with 50 nM of OA effectively abolished both the basal level as well as the TP/HCPT-induced increase in PP2A activity (Fig. 6A). After verifying the inhibition of PP2A activity by OA, we investigated the role of PP2A in the dephosphorylation of ERK1/2, p38 and Akt signaling cascades. As shown in Fig. 6B, OA on its own had no effect on the activation of either ERK1/2 MAPK or p38 MAPK. However, inhibition of PP2A by OA enhanced the ability of TP/HCPT to increase the phosphorylation of ERK1/2 and p38, indicating that the dephosphorylation of the ERK and p38 MAPK signaling pathways might be PP2A-dependent. Despite differential involvement of MAPKs and Akt survival signaling cascades upon treatment with TP and HCPT, OA also effectively prevented Akt dephosphorylation induced by TP/HCPT (Fig. 6B), indicating all the ERK1/2 MAPK, p38 MAPK and Akt signaling cascades might be downstream of PP2A.

Next, to further identify the role of PP2A and its substrates in mediating TP/HCPT-induced apoptosis, we used specific pharmacological inhibitors of the different pathways to evaluate the apoptotic rates. As shown in Fig. 6C, OA pretreatment greatly abrogated the apoptotic effect of TP/HCPT, implying a role for PP2A in TP/HCPT-stimulated apoptosis. PD98059, an inhibitor of MEK1, which is the kinase responsible for the activation of ERK1/2 MAPK, significantly reduced the synergistic effect of TP and HCPT on apoptosis. Similarly, pretreatment of cells with SB203580, a well-established inhibitor of p38 MAPK, also inhibited the apoptotic effect of TP/HCPT, suggesting the activation of ERK and p38 are required for apoptosis caused by TP/HCPT. We then employed LY294002, a well-known PI3K inhibitor, to examine whether the PI3K/Akt signaling pathway also plays a key role in mediating apoptosis. Our data showed that, contrary to the effect observed with the ERK and p38 inhibitors, LY294002 dramatically potentiated TP/HCPT-stimulated apoptosis by 40%, indicating that inhibition of the PI3K/Akt signaling pathway was critical to the combinational effect of TP and HCPT on apoptosis. Together these results indicate that TP/HCPT trigger apoptosis plausibly by activation of the p38 and ERK MAPK cascades, and inhibition of the Akt survival pathway through a mechanism involving activation of PP2A.

## Discussion

Recently, the interest in exploiting traditional Chinese medicine (TCM) for prevention or treatment of cancer has been greatly increased (4). Among TCMs, two well-known herbal medicines triptolide (TP) and hydroxycamptothecin (HCPT), that are produced mainly in regions of China (as well as some other Asian areas such as Japan and North Korea), have been found to exhibit anticancer potential both *in vitro* and *in vivo* (16,43,44). Our previous study, which determined the effects of TP and HCPT on pancreatic cancer, demonstrated that TP combined with HCPT synergistically increase their cytotoxic effect in pancreatic cancer cells PANC-1, suggesting that the combination of TP and HCPT may possess clinical potential for the treatment of cancers (25). As yet, however, the molecular basis underlying the synergistic cytotoxicity of these two herbal medicines has remained poorly understood. We attempted to investigate the efficacy of combinatorial TP/HCPT treatment in human non-small cell lung cancer (NSCLC) cell line A549 *in vitro*. In the present study, the results present an extension of our prior findings by showing that the synergistic TP/HCTP anticancer effect in A549 lung adenocarcinoma cells is mediated by apoptosis induction via activation of mitochondria-dependent apoptotic pathway, along with enhanced PP2A activity as well as activation of ERK1/2 and p38 MAPKs cascades and inhibition of Akt survival pathway, which provide new insight into the mode of action of the traditional Chinese medicine TP together with HCPT in cancer therapy.

A wealth of data indicate that TP, with its broad-spectrum anticancer activity, can be used as a single agent to treat different tumors. Recently, several pieces of evidence have been reported indicating the possibility of using TP in combination with other anticancer drugs to improve efficacy; also as a single drug, TP has been found to enhance the action of other anticancer agents or therapies, such as idarubicin (19), sorafenib (20), 5-FU (21) and ionizing radiation (24), making the combination superior to mono-therapy alone. In addition to the aforementioned agents, our previous study also demonstrated TP combined with HCPT have therapeutic potential for pancreatic cancer (25). Thus, elucidating the mode of action of TP together with HCPT in killing cancer cells will help us to understand and to better use TCMs in cancer therapy. Our present study by keeping TP concentration at its IC_10_ value combined with variable concentrations of HCPT demonstrated a significant decrease in IC_50_ of HCPT which indicated the increased cytotoxicity of HCPT by TP, suggesting that the combinatorial TP/HCPT drug regimens substantially suppressed A549 cell growth as compared to either mono-therapy. Further CI analyses revealed a synergistic interaction between the two agents in most of the combination doses tested. Similar to our recent findings in pancreatic cancer cell line PANC-1 (25), a higher synergistic effect (lower CI value) was observed for lower dose combination. Moreover, combinatorial TP/HCPT treatment yielded favorable DRI values which allowed for both TP and HCPT dose reduction. Taken together, such synergistic interactions between TP and HCPT provide an opportunity to reduce the doses of the individual drug and thereby reducing their adverse toxicities.

Apoptosis (programmed cell death), is characterized by several morphological and biochemical events (45). To determine whether the decrease in A549 cells growth is attributed to apoptosis seems vital, as the induction of apoptosis in cancer cells is the major indicator of anticancer effects. Hereby, Annexin V/PI staining-based FACS analysis was employed to evaluate the proportion of apoptotic cells. Apoptotic cells are characterized by a series of morphological alterations such as shrinkage of the cells and the nuclei, loss of adhesion to adjacent cells, membrane blebbing, DNA fragmentation and chromatin condensation (46). In the present study, A549 cells treated with combinatorial TP/HCPT treatment exhibited evidence of apoptotic morphology, i.e., cellular shrinkage, cytoplasmic blebbing and condensation of nuclei, which are characteristics of apoptosis (data not shown). These observations were confirmed by flow cytometry analysis which clearly revealed the number of apoptotic cells dramatically increased after combined exposure, suggesting that TP acts synergistically with HCPT in promoting apoptosis which greatly contributed to the inhibition of NSCLC A549 cell growth.

Three major distinct pathways have been reported to mediate TP-induced apoptosis in various cell lines, including the death receptor-mediated (extrinsic), mitochondrial-mediated (intrinsic) and the endoplasmic reticulum (ER) stress pathway (11,12,23,47). Mitochondria are known to play a crucial role in the apoptotic cell death induced by anticancer agents (48–50). In the present study, we did observe that the combined treatment of TP and HCPT induced activation of caspase-3, -9 and release of cytochrome *c* from mitochondria into the cytosol, which are regarded as hallmarks of the mitochondria-mediated apoptotic pathway. Moreover, we found that combinatorial TP/HCPT drug regimens resulted in a dramatic increase in expression of pro-apoptotic protein Bax, while the expression of anti-apoptotic protein Bcl-2 was markedly inhibited, suggesting a shift in the dynamic balance between the outputs of pro-apoptotic and anti-apoptotic pathways. Based upon the fact that the Bcl-2/Bax ratio plays a crucial role in cancer cell apoptosis (51), we reasoned that the reduction in Bcl-2/Bax ratio by TP/HCPT would allow less Bcl-2-Bax complex. The net effect would be the release of more free Bax, which then translocated into the mitochondrial membrane and induced the opening of the mitochondrial permeability transition pore to allow the release of cytochrome *c* and ultimately triggered the caspase cascade activation. There is emerging evidence for a close ER-mitochondria relationship (52), and cross-talk between mitochondrial and ER plays an essential role in determining cell commitment to apoptosis (53,54). Therefore, we further investigated the hallmarks of ER stress-mediated apoptosis, including glucose-regulated protein 78 (GRP78) and its downstream pro-apoptotic proteins CHOP and caspase-12. GRP78 has been documented as a key regulator in ER stress signaling that has a dynamic capacity to regulate the balance between cell survival and apoptosis in ER-stressed cells (29). TP mono-therapy has been reported to downregulate GRP78 and leads to ER stress-mediated apoptosis in pancreatic cancer cells (12). Intriguingly, in stark contrast, our study presented herein revealed that TP/HCPT combination treatment upegulated the expression of GRP78, while the downstream proteins CHOP and caspase-12, as the critical executioners involved in ER stress-mediated apoptosis (30,31), were not affected. It is possible that in the early stage of ER stress, the GRP78 was activated by TP/HCPT to alleviate the stress, thereby restoring the ER homeostasis and leading to prevention of the induction of downstream CHOP and caspase-12. Taken together, these findings suggest that combinatorial TP/HCPT drug regimens induce caspase-dependent apoptosis in A549 lung cancer cells mainly through modulating the Bax- and Bcl-2-triggered mitochondrial pathway.

Many studies have been reported that the pro-apoptotic activity of TP is due to its modulation of apoptosis-activating proteins (10,47,55). It appears from these studies that TP influences multiple proteins and pathways associated with cell growth and survival. Likewise, many studies demonstrate that HCPT can induce apoptosis of various cancer cells by influencing the expression of cancer suppression genes. The direct targets of TP together with HCPT, however, remain unidentified. Numerous studies have reported that MAPKs and Akt/PKB signal transduction pathways play crucial roles in a variety of chemotherapeutic agent-induced apoptotic signaling (33,34). The three distinct sets of MAPKs are known to be activated differentially depending on the cell type and the nature of the stimuli administered (56,57). Our study demonstrated that the combined treatment of TP and HCPT exhibited an enhancing effect on both the phosphorylation of ERK and p38. Of the MAPKs, ERK1/2 behaves as a mitogen-activated factor that is believed to mediate both cell proliferation and survival, whereas p38 and JNK are activated in response to cellular stresses and appear to exert both protective as well as pro-apoptotic effects (57–59). Intriguingly, in the present study, pretreatment of the cells with either ERK-inhibitor PD 98059 or p38-inhibitor SB 203580 could effectively abolished the apoptotic effect of TP/HCPT, indicating that the activation of mitogenic stimuli-activated ERK and stress-induced p38-MAPK both contribute to TP/HCPT-induced apoptosis. Next, we tried to characterize the phosphorylation status of Akt which is known as an anti-apoptotic kinase (34). In the present study, we found that TP/HCPT triggered remarkable downregulation of Akt pathway. Moreover, contrary to the effect observed with the ERK and p38 inhibitors, inhibition of the Akt pathway with the PI3K inhibitor LY294002 potentiated TP/HCPT-induced apoptosis in A549 cells, which is consistent with the notion that the Akt cascade provides survival signals that counter-balance the apoptotic response induced by TP/HCPT. Thus, despite differential involvement of MAPKs and Akt survival signaling cascades upon treatment with TP and HCPT, the activation of ERK1/2 and p38, and inhibition of Akt signal transduction pathway could plausibly all contribute to eliciting apoptosis in A549 cells. However, the potential of cross-talk between the MAPKs and Akt pathways still needs further investigation.

Recently, protein phosphatase 2A (PP2A), an important protein serine/threonine phosphatase, is attracting more and more attention due to its apoptosis-inducing effect and tumor-suppressing function (36–38). In the present study, we observed that TP/HCPT stimulated a significant enhancement of PP2A activity, along with alterations of its composition subunits, including down-regulated PP2A catalytic C subunit, up-regulated PP2A structural A subunit, and decreased phosphorylation at PP2A-C Tyr307 (Y307). PP2A-A subunit, a scaffolding protein for the holoenzyme, is reported to allosterically modulate the enzymatic properties of PP2A-C (60), we thus reasoned that enhanced expression of PP2A-A might contribute to the induction of PP2A activity. Previous studies have revealed that post-translational modifications of the highly conserved carboxy-terminal sequence of PP2A-C subunit, especially the phosphorylation at Tyr307 can affect PP2A phosphatase activity (35,39). In the present study, we found the depression of phosphorylation at PP2A-C Y307 closely matched the enhancement of PP2A activity.

PP2A is one of the most studied regulators of MAPKs and Akt by dephosphorylating the threonine or the tyrosine residue and thereby downregulate their activities (32,40), and is known to directly interact with these kinases (41). Our previous study demonstrated that PP2A inhibition, by its specific inhibitor MC-LR, leads to a dramatic activation of p38-MAPK (28). In agreement with the prior findings, the present study demonstrated the downregulation of p38 and ERK MAPKs by PP2A, as a specific PP2A inhibitor of OA resulted in a significant increase in TP/HCPT-induced phosphorylation of ERK1/2 and p38. On the other hand, OA dramatically reversed the down-regulation of Akt phosphorylation, suggesting the involvement of a phosphatase 2A in TP/HCPT-induced dephosphorylation of Akt. However, our present study also showed that a significant increase in PP2A activity triggered by TP/HCPT was surprisingly accompanied by abnormal phosphorylation of ERK and p38, implying that the modulation of the MAPK signaling pathway is not all in a phosphatase-dependent manner. Actually, because the phosphorylation of MAPKs is a reversible process which is regulated by a coordinated balance between protein kinase-mediated phosphorylation and protein phosphatase-mediated dephosphorylation, it might be possible that, in addition to PP2A activation, TP/HCPT stimulate the activation of ERK- and p38-MAPK also by acting on the MAPKs upstream kinases, for example, MAPK kinases (MKKs) such as MKK1/2 (ERK upstream kinases) and MKK3/6 (p38 upstream kinases), and even the MKK upstream kinase MAPK kinase kinases (MKKKs) (61), which requires further research.

**Figure 7:**
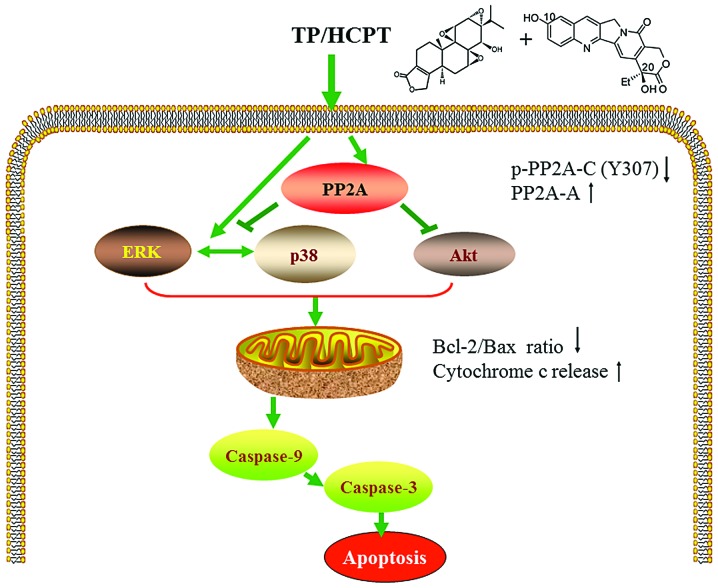
Schematic diagram of the mechanism by which TP together with HCPT induce apoptosis in A549 lung adenocarcinoma cells. TP and HCPT synergistically stimulate the activation of tumor suppressor PP2A, and modulate PP2A-regulated intracellular signaling pathways, involving activation of ERK1/2 and p38 MAPKs cascades, and inhibition of Akt survival pathway. Finally, combinatorial TP/HCPT drug regimens cause Bax- and Bcl-2-mediated mitochondrial apoptotic pathways, resulting in caspase-9 and -3 triggered cell apoptosis.

In conclusion, our study discovers a new mode of action of TP together with HCPT in anticancer therapy. To our knowledge, this is the first report demonstrating that TP and HCPT synergistically exert *in vitro* anticancer activity in human NSCLC A549 lung adenocarcinoma cells through induction of apoptosis. Importantly, our findings suggest that TP/HCPT trigger apoptosis in human lung cancer cells by activation of p38 and ERK MAPK cascades, and inhibition of the Akt survival pathway through a mechanism involving activation of PP2A, which uncover a molecular mechanism that may underlie this combinatorial therapy (Fig. 7). Additionally, such synergistic interactions, which allow for both TP and HCPT dose reduction, raise the interesting possibility that TP/HCPT may have a clinically beneficial effect on anticancer dose reduction and, thereby leading to a decrease in cytotoxicity during chemotherapy. Since our present conclusions were primarily based on *in vitro* studies, *in vivo* assessment of the anticancer effect of TP/HCPT in e.g., xenograft models is warranted. Further studies are required to improve our understanding of the synergistic anticancer action of TP/HCPT and to develop new combinatorial therapies for cancer.

## Figures and Tables

**Table I:** Combination index (CI) and dose reduction index (DRI) analysis for triptolide and hydroxycamptothecin combination therapy in A549 cells.

Combination therapy				
			
HCPT (μg/ml)	TP (ng/ml)	CI	DRI for HCPT	DRI for TP
0.5	25	1.18	3.74	1.10
1	25	0.46	4.51	4.21
2	25	0.40	3.52	8.34
4	25	0.41	2.84	17.39
8	25	0.67	1.60	20.97

The affected fraction of cell survival after drug treatment is calculated with respect to control, and CalcuSyn software was run with relevant data as per in Materials and methods, and the CI and DRI values of the different combinations is computed. CI determine the type of interaction [additive (CI=1); synergistic (CI<1); antagonistic (CI>1)]. The DRI determines the magnitude of dose reduction allowed for each drug when given in synergistic combination, as compared with the concentration of a single agent that is needed to achieve the same effect.
